# Biomolecule screen identifies several inhibitors of *Salmonella enterica* surface colonization

**DOI:** 10.3389/fbioe.2024.1467511

**Published:** 2025-01-03

**Authors:** Joseph Headrick, Amital Ohayon, Shannon Elliott, Jacob Schultz, Erez Mills, Erik Petersen

**Affiliations:** ^1^ Department of Biomedical Health Sciences, College of Public Health, East Tennessee State University, Johnson City, TN, United States; ^2^ Department of Animal Sciences, Robert H. Smith Faculty of Agriculture, Food, and Environment, The Hebrew University of Jerusalem, Rehovot, Israel; ^3^ Department of Environmental and Occupational Health and Safety Sciences, College of Public Health, East Tennessee State University, Johnson City, TN, United States

**Keywords:** *Salmonella*, surface colonization, salicylic acid, biomolecule, eggshell

## Abstract

*Salmonella enterica* is a foodborne pathogen commonly found in agricultural facilities; its prevalence, as well as increasing levels of disinfectant- and antibiotic-resistance, has significant costs for agriculture as well as human health. In an effort to identify potential new inhibitors of *S. enterica* on abiotic surfaces, we developed a biomolecule screen of nutrient-type compounds because nutrients would have lower toxicity in animal facilities and bacterial nutrient utilization pathways might prove less susceptible to the development of bacterial resistance. After screening 285 nutrient-type compounds, we identified ten that significantly inhibited the ability of *S. enterica* to colonize a plastic surface. After conducting a dose-response curve, salicylic acid was selected for further testing due to its low minimal inhibitory concentration (62.5 μM) as well as a low total inhibitory concentration (250 μM). Salicylic acid was also able to inhibit surface colonization of a wide range of bacterial pathogens, suggesting that our biomolecule screen might have broader application beyond *S. enterica*. Finally, we determined that salicylic acid was also able to inhibit *S. enterica* colonization of an organic surface on eggshells. Together, these results suggest that nutrient-type biomolecules may provide an avenue for preventing resistant bacteria from contaminating surfaces.

## 1 Introduction

Non-typhoidal *Salmonella enterica* is a zoonotic pathogen with multiple serovars, including several that are major causes of foodborne illness (Gal-Mor et al., 2014; Galán, 2021). Of the serovars that account for human infections, *S. enterica* serovars Enteritidis and Typhimurium are the two primary culprits (Bawn et al., 2020; Shaji et al., 2023). Often, these foodborne infections occur through the contamination of poultry meat or other poultry products like eggs (Humphrey and Jørgensen, 2006; Ehuwa et al., 2021). This contamination occurs due to the persistence of *Salmonella* within poultry and their facilities, resulting in high costs to both infected humans and poultry hosts (Aljumaah et al., 2020; Scharff, 2020).

Current methods to clear *Salmonella* from poultry facilities include chlorine-containing compounds like bleach, quaternary ammonium chloride chemicals, and formaldehyde, but these have several limitations (Obe et al., 2024). Chlorine-based disinfectants and formaldehyde to remove *Salmonella* from surfaces can be toxic to the poultry and workers in the same facility (Iji et al., 2013; Keïta et al., 2016; Oliveira et al., 2020). Furthermore, the use of quaternary ammonium compounds and other chemical disinfectants is leading to the emergence of resistant bacteria, necessitating further strategies (Wessels et al., 2021; Ohashi et al., 2022; Papić et al., 2022). The antibiotic treatment of chickens can contain and limit infections, but this also comes at both a financial and biological cost and is leading to further resistance within pathogen populations (Graham et al., 2007; Ferreira et al., 2019; Castro-Vargas et al., 2020; Abreu et al., 2023). These then necessitate the identification and study of new compounds to help combat *Salmonella* infections within poultry facilities.

Rather than testing synthetic chemicals or novel antibiotics to combat *Salmonella,* we sought to investigate nutrient-type biomolecules for their ability to inhibit bacterial colonization of surfaces. Such biomolecules were selected because these compounds are less likely to be toxic to animals and humans within the treatment area, and bacteria may have a reduced capacity to develop resistance to their presence compared to other chemicals that do not provide a nutritional benefit. To identify biomolecules with inhibitory activity against *S. enterica*, we developed a 96-well plate-based semi-high throughput screen of nutrient-type compounds.

## 2 Materials and methods

### 2.1 Media and growth conditions

*S. enterica* serovar Typhimurium strain 14028s was our primary bacterial strain. Cultures for biomolecule testing were typically grown in 2 mL of M63 defined medium overnight for 20–24 h at 37 °C with shaking at 250 rpm. M63 medium contained 0.5X M63 salts (21 mM dibasic potassium phosphate, 11 mM monobasic potassium phosphate, and 4.4 mM ammonium sulfate final concentration), 1X Corning^®^ MEM essential amino acids, 1X Corning^®^ MEM nonessential amino acids, 0.23% glycerol, 2 mM MgCl_2_, 1 mM NaCl, and 10 µM FeCl_3_. Lysogeny broth (LB) was also used as a standard culture condition for growth rate assays. LB media consisted of 10.0 g/L Fisher BioReagents™ tryptone, 5.0 g/L Fisher BioReagents™ yeast extract, and 10.0 g/L Fisher BioReagents™ NaCl. LB agar plates consisted of the LB components as well as 15 g/L Fisher BioReagents™ bacteriological agar. The strains used were stored indefinitely at −80 °C in their original growth medium with 20% glycerol for preservation. All water used was autoclaved Milli-Q ultrapure water.

### 2.2 Plate reader growth rate assay for bacterial presence

*S.* Typhimurium was grown in M63 media with amino acids for 20–24 h at 37 °C to reach the stationary phase. To remove any residual growth media that could result in osmotic pressure upon the bacteria, the overnight culture was centrifuged at 15,000 rpm, washed thrice with water, and resuspended to an OD_600_ of 1.0 (approximately 1 × 10^9^ colony-forming units [CFU]/mL). From this 1.0 OD_600_ dilution, six serial five-fold dilutions were produced in water. We arrayed 10 μL of each diluted sample and a water blank in a 96-well plate (n = 3), and 200 µL of LB was pipetted into each well to allow for bacterial growth. The plate was incubated in a BioTek^®^ Synergy HTX Multimode Reader at 37 °C with OD_600_ reads every 5 min following 30-s orbital shake periods and was analyzed with Gen5™ v3.11 Microplate Reader and Imager software. Linear regression of the time to OD_600_ = 0.5 (mid-log) *versus* the predicted CFU/mL were used to determine an approximated CFU/mL equation.

### 2.3 Biomolecule screen for inhibition of surface colonization

The biomolecule screen consisted of Biolog Phenotype Microarray™ plates PM1, PM2A, and PM3B (Bochner et al., 2001). Phenotype Microarray plates were prepared by resuspending the biomolecules in 100 µL of water, shaking the plate at 1,000 rpm for 10 min, incubation at 37 °C for 30 min, then shaking the plate again at 1,000 rpm for 10 min. The Phenotype Microarray plates were stored at −20 °C after each trial. *S.* Typhimurium was grown in M63 media for 20–24 h at 37 °C to reach the stationary phase, washed with water as described above, and diluted to 2.0 OD_600_ in water.

**FIGURE 1:**
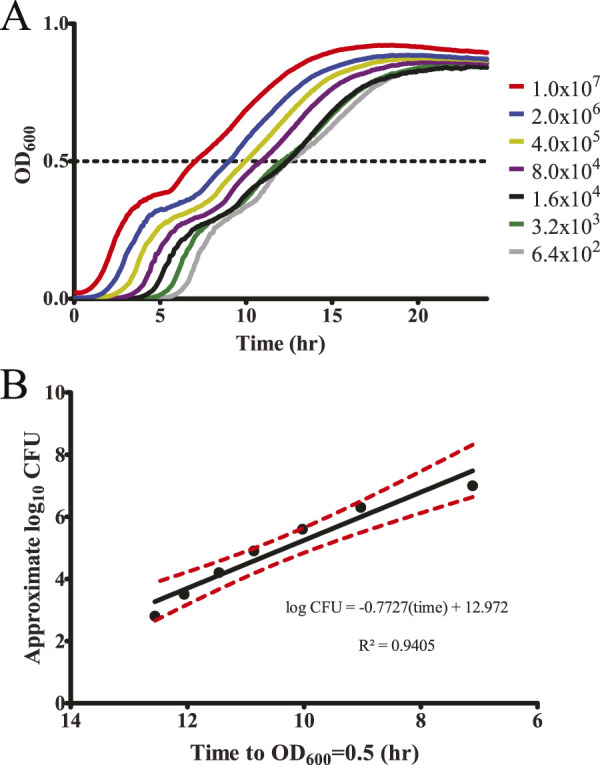
Plate reader growth assay accurately monitors starting bacteria concentration. **(A)** To evaluate a plate reader-based growth assay to detect changes in surviving bacteria within a well, a dilution series of *S.* Typhimurium was generated based on the expected CFU as determined by OD_600_ (assuming 1.0 OD_600_ = 1 × 10^9^ CFU/mL). These samples were grown overnight within a plate reader incubated at 37 °C with shaking and OD_600_ measurements every 5 min n = 3. **(B)** Growth to a mid-log point (OD_600_ = 0.5) is an accurate measurement of differences in starting bacterial concentration as graphed *versus* the expected CFU/mL, allowing us to approximate the number of surviving bacteria based on time to OD_600_ = 0.5. Red dashed lines indicate 95% confidence interval.

Surface colonization assays were prepared by mixing 5 µL of each biomolecule and 5 µL of the 2.0 OD_600_ sample of *S.* Typhimurium in a 96-well plate. The bacteria were allowed to completely dry in a running biosafety cabinet for 2–3 h, then the surface colonization plate was incubated for 20–24 h at 25 °C. Surface colonization was monitored by resuspending surviving bacteria in 200 μL LB. Growth within plates was monitored via a plate reader as described above. The time to OD_600_ = 0.5 was determined for exposure to each nutrient. Wells that failed to reach an OD_600_ of 0.5 over the course of the experiment were assigned a value of 18 h for statistical calculations. Each Phenotype Microarray plate was tested in triplicate. To test for statistical significance against the no-biomolecule negative control, the results were analyzed alongside the similar compounds within each Phenotype Microarray row with repeated-measures ANOVA with Dunnett’s multiple comparison test (Supplementary Table S1). Approximate CFU/mL values were calculated according to the equation determined above (Figure 1B).

To ensure that we were not detecting general inhibitory effects from these biomolecules during the LB growth analysis, growth inhibition assays were prepared by filling a 96-well plate with 190 µL of LB, 5 µL of each biomolecule and 5 µL of the 2.0 OD_600_ sample of *S.* Typhimurium. Samples were tested as described above in triplicate, and the average time to OD_600_ = 0.5 was determined. Surface colonization values were normalized to the growth inhibition values, and these were both normalized to their respective water-only controls (100%) to confirm that results were indicative of inhibited surface colonization (Supplementary Table S2).

### 2.4 Individual biomolecule surface colonization inhibition assay

Inhibitory biomolecules identified in the screen were separately purchased and tested for the inhibition of surface colonization under a range of concentrations. We prepared 10 mM stocks in water of each chemical, then diluted them to 2 mM, followed by a two-fold serial dilution in water. After factoring in the two-fold dilution from bacterial addition, these resulted in final biomolecule concentrations of 1,000, 500, 250, 125, 62.5, and 31.25 μM to be tested alongside a water-only negative control. In the stationary phase, washed *S.* Typhimurium was prepared as described above for the biomolecule screen. We mixed 5 μL of this 2.0 OD_600_ bacterial suspension with 5 μL of the specified biomolecule dilution and processed as above. The assay was repeated in quadruplicate. Surface colonization inhibition values were analyzed against the water-only negative control with one-way ANOVA with Dunnett’s multiple comparison test for statistical significance.

### 2.5 Colony-forming unit measurement of surface colonizing bacteria

To confirm that our plate reader growth assay was accurately monitoring surviving bacteria, we repeated the concentration curve described above for salicylic acid. The washed stationary phase *S.* Typhimurium was exposed to the salicylic acid concentration curve and a water-only negative control within the first column of a 96-well plate. After drying and allowing surviving bacteria to colonize the surface of the 96-well plate for 24 h, these bacteria were resuspended by adding 200 µL of 1X PBS to inoculated wells, and the 96-well plate was shaken at 1,000 rpm for 20 min to rehydrate and resuspend surviving bacteria.

Colony-forming units (CFUs) of surviving surface colonizing bacteria were determined by conducting a five-fold dilution series within the 96-well plate and quantifying it through a 6 × 6 drop plate method (Chen et al., 2003). Briefly, 160 μL of 1x PBS was added to columns 2–12 of the 96-well plate. The bacterial resuspension in column 1 was five-fold serially diluted by the sequential transfer of 40-μL aliquots to each successive well. We then pipetted 7-μL spots of each well in the dilution series in sextuplet onto LB plates and allowed them to dry. These plates were incubated overnight at 25 °C followed by a brief 37 °C the following day until the colony size was countable. Colony counts from a single dilution were determined and averaged across the six replicate spots to determine CFUs. The assay was repeated in triplicate. Results were analyzed against the water-only control with one-way ANOVA with Dunnett’s multiple comparison test for statistical significance.

### 2.6 Salicylic acid testing for surface colonization against other bacterial pathogens

To determine whether the inhibition of surface colonization by salicylic acid was specific to *S.* Typhimurium or more broadly applicable, several other bacterial pathogens were tested for surface colonization under a concentration curve of salicylic acid. *Acinetobacter baumannii* strain ATCC17978, *Citrobacter freundii* strain ATCC8090, *Escherichia coli* strain MG1655, *Klebsiella pneumoniae* strain ATCC13883, *Pseudomonas aeruginosa* strain PA14, *Proteus vulgaris* strain ATCC13315, *Staphylococcus aureus* strain ATCC25923, and *Serratia marcescens* strain ATCC13880 were processed identically as described above and tested for surface colonization inhibition by the salicylic acid concentration curve using the plate reader growth assay. This experiment was repeated in quadruplicate and statistically analyzed against the water-only negative control for each strain with one-way ANOVA and Dunnett’s multiple comparison test for statistical significance.

### 2.7 Eggshell surface colonization assay

We designed an eggshell surface colonization assay to investigate whether salicylic acid would also inhibit the surface colonization of an organic surface by *S.* Typhimurium. Eggshells were prepared by cracking commercial washed eggs in half and emptying their internal contents into waste within a biosafety cabinet. The two halves of the eggshells were then placed facing upward and allowed to dry for 15 min under the biosafety cabinet airflow. Using sterilized tweezers, 1 × 1 cm eggshell fragments were separated. These fragments were placed in one-half of a 24-well plate with the exterior half of the fragment facing upward, leaving the other half of the 24-well plate empty for resuspension of the bacteria after desiccation. These eggshell fragments were allowed to sit overnight to finish drying.

*S.* Typhimurium was grown to stationary phase and washed as described above, then diluted with either water, salicylic acid, or l-arginine to a final concentration of 1.0 OD_600_ bacteria and 250 μM biomolecule in a final volume of 100 µL in microcentrifuge tubes. We pipetted 10 µL of each sample with additive onto the eggshell fragments within the 24-well plates. The bacteria were allowed to dry in a biosafety cabinet for 2–3 h, then the eggshell fragments were incubated for 20–24 h at 25 °C. Each eggshell fragment was inverted into 500 µL of 1x PBS in the other half of the 24-well plate, and the plate was shaken at 1000 rpm for 20 min to allow surviving bacteria to resuspend within the 1x PBS. The CFUs of the surviving bacteria were calculated using the 6 × 6 drop plate method described above. The survival of each additive was quantified by calculating its sample’s relative survival percent change compared to the water-only control. Each trial consisted of triplicate eggshell samples for each biomolecule from one egg. An additional no-bacteria control without *S.* Typhimurium was used to ensure that the eggshells were not initially contaminated with bacteria. The entire assay was repeated in triplicate, and the results were analyzed with a paired *t*-test against the water-only control for statistical significance.

## 3 Results

### 3.1 Several nutrient-type biomolecules inhibit the ability of *Salmonella* Typhimurium to colonize surfaces

To screen nutrient-type biomolecules for inhibitory activity against surface colonization by *S. enterica* serovar Typhimurium, we utilized Biolog Phenotype Microarray plates to enable a semi-high-throughput screen of organic nutrients (Bochner et al., 2001). These plates are typically used for identifying nutrient utilization pathways in particular bacteria, but we found them to be a convenient source of ordered nutrients within a 96-well format. We selected 285 nutrients from plates PM1–3 containing carbon and nitrogen source nutrients—including a range of carbohydrates, amino acids, organic acids, and other metabolites—for analysis. A screening technique was then developed to determine whether these chemicals could inhibit *S.* Typhimurium from colonizing a surface. To monitor bacterial survival, a plate-reader-based growth assay was used to monitor kinetic growth over time until a mid-log growth point (OD_600_ = 0.5) (Figure 1A). This also enabled us to approximate the number of surviving CFUs prior to growth based upon growth time to mid-log (OD_600_ = 0.5) (Figure 1B). While this approximation comes with some caveats, such as differences in lag phase after chemical exposure, altered replication rates under exposure to a particular biomolecule, it allowed us to better visualize the inhibitory effect during our screen.

*S.* Typhimurium was grown to the stationary phase, washed to remove residual culture medium that could provide osmotic pressure as the bacteria are dried onto a surface, and mixed with the nutrient-type biomolecules in water alongside a water-only negative control. These treated *S.* Typhimurium samples were allowed to colonize a plastic surface through drying and incubated for 20 h at 25 °C. To quantify the bacteria that survived treatment, these samples were regrown in LB, and the time until the mid-log growth point was determined via kinetic density measurements. These time-to-mid-log values were converted to approximate the log_10_ change in CFU after biomolecule treatment.

After testing these 285 nutrient-type biomolecules for their ability to inhibit the colonization of surfaces by *S.* Typhimurium and adjusting for any growth-phase specific inhibition (Supplementary Table S1), we identified ten biomolecules that significantly inhibited surface colonization with at least a one log_10_ decrease in approximate CFU/mL (Table 1). These inhibitory nutrient-type biomolecules included a carbohydrate, a nucleotide, and several small organic acids. To ensure that we were detecting an inhibition of surface colonization and not an inhibition during regrowth in LB, we also conducted a growth assay of *S.* Typhimurium exposed to each of the biomolecules in our screen (Supplementary Table S2). None of the identified biomolecules displayed growth-phase inhibition, suggesting that their inhibitory activity was specifically occurring during *S.* Typhimurium surface colonization.

**TABLE 1:** Biomolecule inhibitors of surface colonization by *S.* Typhimurium.

Chemical	Approximate log_10_ CFU change^a^	Significance^b^
Dihydroxyacetone	−4.40 log_10_	***
2-Deoxy-d-Ribose	−3.97 log_10_	***
Itaconic acid	−3.95 log_10_	***
Alloxan	−3.16 log_10_	**
Guanine	−2.83 log_10_	*
Sorbic acid	−2.68 log_10_	**
Glyoxylic acid	−2.47 log_10_	***
α-Hydroxybutyric acid	−2.30 log_10_	**
p-Hydroxyphenylacetic Acid	−2.12 log_10_	**
Salicylic acid	−1.72 log_10_	**

^a^ The change in log_10_ CFU values was approximated using the linear regression from Figure 1B.

^b^ Significance was calculated using repeated-measures ANOVA, with Dunnett’s multiple comparison test against the water-only negative control.

### 3.2 Identified biomolecules display a range of inhibitory concentrations

**FIGURE 2:**
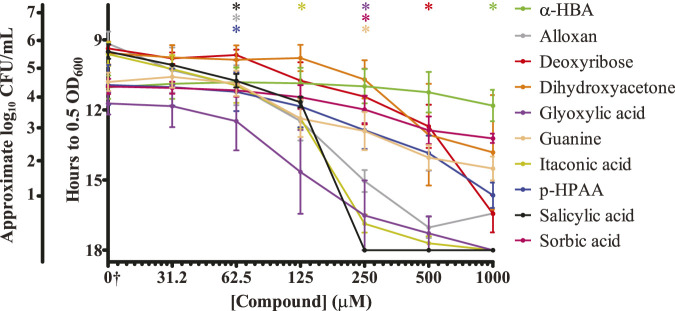
Identified biomolecules inhibit the ability of *S.* Typhimurium to colonize surfaces. Biomolecules identified through the nutrient-type screen were analyzed for their ability to inhibit surface colonization by *S.* Typhimurium at a range of concentrations. Washed stationary phase *S.* Typhimurium was exposed to each of the indicated concentrations, dried upon a plastic surface, and incubated at 25 °C for 20 h. LB growth media was then added to each sample, and regrowth of any surviving bacteria was monitored via plate reader for 18 h. The *y*-axis is graphed inversely for time to indicate that longer times present fewer surviving bacteria. Approximated log_10_ CFU/mL counts are provided alongside the time to OD_600_ = 0.5 for reference. n = 4. Statistical significance was determined using one-way ANOVA with Dunnett’s multiple comparison test against the water-only control, and lowest concentration at which treatment became significant (*p* < 0.05) is displayed along the top of the graph for each sample. Only dihydroxyacetone did not statistically significantly inhibit surface colonization of *S.* Typhimurium at the tested concentrations. †The water-only control for each sample is graphed on the log_2_ *x*-axis as 15.625 to enable visualization (0†).

One drawback to the use of Biolog Phenotype Microarray plates is a variable concentration of different nutrients within the plates. Because these plates are designed to be used as nutrient sources for growth, individual compounds are included within the plates at proprietary concentrations. Furthermore, any future development into a commercial product would likely focus on biomolecules with low effective dose-to-limit side effects. To determine the true effective concentration of our identified inhibitory biomolecules independent of the concentration within the Phenotype Microarray plates, each was separately purchased and tested against *S.* Typhimurium surface colonization. A two-fold dilution series of each biomolecule ranging from 31.25 μM to 1 mM was tested using our plate-reader-based surface colonization assay (Figure 2). While dihydroxyacetone exhibited a high degree of inhibition within the Phenotype Microarray testing, it did not display a statistically significant reduction in surface colonization at concentrations lower than 1 mM. Conversely, salicylic acid displayed both a low statistically significant minimal inhibitory concentration (62.5 μM) as well as the lowest concentration at which no samples reached our growth mid-point (250 μM). For these reasons, salicylic acid was selected for further testing.

### 3.3 Salicylic acid reduces the number of surviving *S.* Typhimurium upon a surface

**FIGURE 3:**
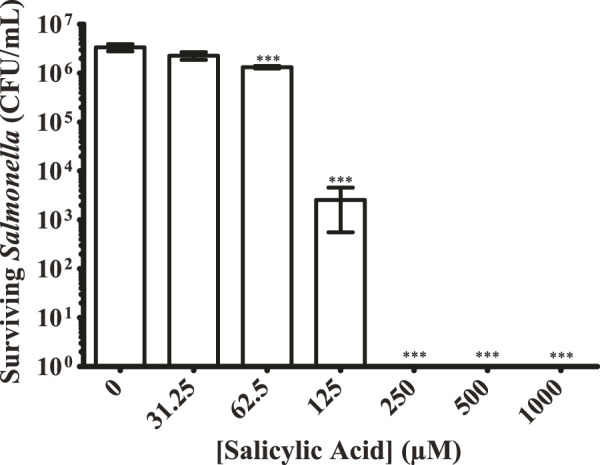
Salicylic acid treatment greatly reduces the number of surviving *S.* Typhimurium present on a surface. *S.* Typhimurium was prepared as before with exposure to a range of salicylic acid concentrations and allowed to colonize a plastic surface. Any surviving bacteria were resuspended in PBS and plated to quantify the CFUs present under each condition. Salicylic acid was able to significantly inhibit surface colonization by *S.* Typhimurium at 62.5 μM, eliminating detected CFUs beginning at 125 μM n = 3. Statistical significance was determined using one-way ANOVA with Dunnett’s multiple comparison test (*** = *p* < 0.001).

While our plate-reader-based growth assay can quantitatively measure the replication time of surviving bacteria, we also wanted to quantitate the number of bacteria surviving on a surface after treatment with salicylic acid. This would also serve as a contrast to the use of our approximate CFU/mL determinations. We prepared the same salicylic acid concentration curve and applied it to *S.* Typhimurium that was then allowed to dry upon a plastic surface. In lieu of adding LB and growing as done previously, we resuspended any surviving bacteria in PBS and plated them to determine the number of surviving CFUs (Figure 3). Just as with the plate reader results, treatment with 62.5 μM of salicylic acid significantly reduced the number of surviving *S.* Typhimurium 2.5-fold, while treatment with 125 μM salicylic acid reduced survival by 3-log, and treatment with 250 μM salicylic acid completely inhibited *S.* Typhimurium. These results are similar to those found using the plate-reader-based growth assay (Figure 2), suggesting this to be a viable method for quantifying bacterial survival.

### 3.4 Salicylic acid inhibits the surface colonization of several different bacterial pathogens

**FIGURE 4:**
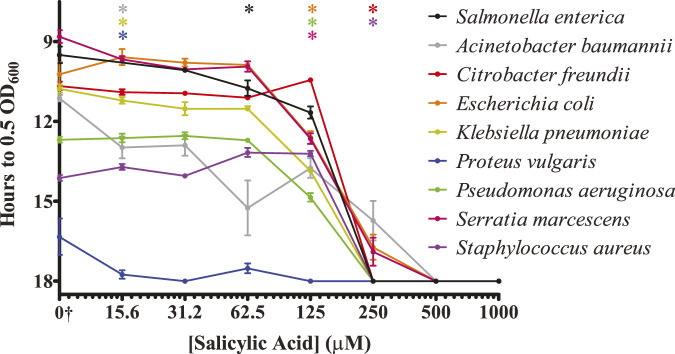
Salicylic acid treatment inhibits the ability of several bacterial pathogens to colonize surfaces. The indicated bacterial pathogens were treated similarly to *S.* Typhimurium and analyzed for the ability of salicylic acid to inhibit their colonization of a plastic surface. Surviving bacteria were monitored via regrowth in culture media via plate reader. The *y*-axis is graphed inversely for time to indicate that longer times present fewer surviving bacteria. n = 4. Statistical significance was determined using one-way ANOVA with Dunnett’s multiple comparison test against the water-only control for each individual organism, and the lowest concentration at which treatment became significant (*p* < 0.05) is displayed along the top of the graph for each organism. All indicated bacterial strains exhibit inhibition of surface colonization by salicylic acid. †The water-only control for each sample is graphed on the log_2_ *x*-axis as 7.8125 to enable visualization (0†).

The purpose of our original screen was to identify nutrient-type biomolecules that could inhibit the colonization of surfaces by *S.* Typhimurium. Having identified several such molecules and focused upon salicylic acid, we decided to investigate whether salicylic acid was similarly able to inhibit other bacterial pathogens or whether this inhibitory action was specific to *S.* Typhimurium. Because these organisms were likely to display a range of growth rates and abilities to colonize plastic surfaces, each organism was exposed to either water or an increasing concentration of salicylic acid. Any inhibitory effect from the salicylic acid was then statistically compared to that organism’s ability to reach our growth midpoint in the water-only exposure control. We were able to detect a significant inhibition in surface colonization by salicylic acid treatment in all organisms tested (Figure 4). Three pathogens (*A. baumannii, K. pneumoniae,* and *P. vulgaris*) displayed a higher sensitivity to salicylic acid treatment, being significantly inhibited at a minimal concentration of 15.6 μM. *A. baumannii* was also a member of a group that was still able to partially survive treatment at 250 μM (also including *C. freundii* and *E. coli*), but none of the tested organisms were able to reach our mid-log growth point after treatment with 500 μM salicylic acid. While only salicylic acid was tested in this assay, this suggests that some of our identified inhibitory biomolecules may have a broad-spectrum activity against bacterial pathogen surface colonization.

### 3.5 Salicylic acid also inhibits *S.* Typhimurium colonization of eggshell surfaces

While we have shown that salicylic acid can inhibit the colonization of an abiotic plastic surface, we also sought to determine whether it could inhibit the colonization of an organic surface. Previously, we had also discovered that salicylic acid inhibits biofilm formation of *S.* Typhimurium through regulating cellulose production (Mills et al., 2015), and we considered whether inhibiting cellulose production was a possible mechanism for salicylic acid’s inhibition of surface colonization. Our previous report identified L-arginine as a stimulator of cellulose production (Mills et al., 2015), so we tested both 250 μM salicylic acid and L-arginine against a water-only control for their ability to alter surface colonization of eggshells, which was then quantified through plating for CFUs.

**FIGURE 5:**
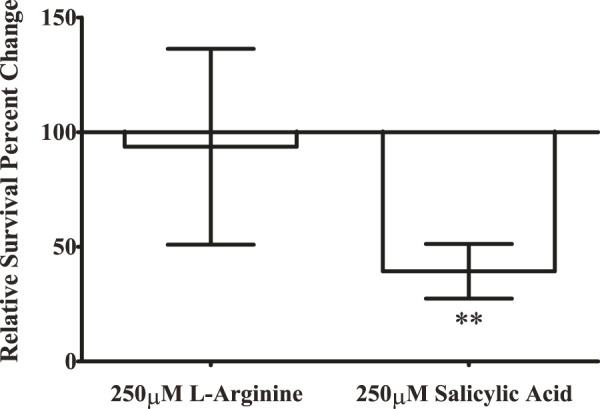
Salicylic acid treatment inhibits the ability of *S.* Typhimurium to colonize the surface of an eggshell. *S.* Typhimurium was prepared as previously and applied to the surface of an eggshell for surface colonization under treatment with either 250 μM salicylic acid or a water-only control. To determine whether the ability of salicylic acid to inhibit cellulose production is involved in this phenotype (Mills et al., 2015), we also tested the cellulose-inducer L-arginine at 250 μM. After allowing *S.* Typhimurium to colonize for 20 h, surface-colonized bacteria were resuspended from the surface of the eggshell fragments with PBS and plated to quantify CFUs of surviving bacteria. A no-bacteria control was included in each experiment to ensure the eggshell fragments were free of detectable contamination. Each experiment consisted of three technical replicates upon individual eggshell fragments, and these three CFU values were averaged for a single data point. The entire experiment was repeated three times (n = 3) and results were analyzed with paired *t*-test against the water-only control for statistical significance. (** = *p* < 0.01).

We measured a significant reduction in the number of bacteria able to colonize the surface of an eggshell during salicylic acid treatment (Figure 5). However, treatment with the cellulose production stimulator L-arginine did not produce any significant change in surface colonization. Furthermore, salicylic acid treatment of a mutant in the cellulose synthase gene *bcsA* replicates the inhibitory effect of salicylic acid (data not shown), suggesting that altering cellulose production is not responsible for the inhibition of surface colonization by salicylic acid. However, this does indicate that salicylic acid may have an inhibitory effect on a number of surfaces that could be colonized by *S.* Typhimurium.

## 4 Discussion

The goal of this project was to identify potential biomolecules that could disrupt *Salmonella* colonization of surfaces to serve as a disinfection option either alone or in combination with other compounds. A screen of 285 nutrient-type biomolecules derived from carbon and nitrogen nutrient-type compounds identified ten biomolecules with inhibitory activity against *S. enterica* colonizing a plastic surface. Of the potential inhibitors, six are classified as organic acids—salicylic, itaconic, glyoxylic, sorbic, p-hydroxyphenylacetic, and α-hydroxybutyric acids. Organic acids have been established as feed additives for chickens due to their apparent protection against pathogens, promotion of broiler growth, and enhancement of feed efficiency (EFSA Panel on Additives and Products or Substances used in Animal Feed, 2015; Alagawany et al., 2017; Pearlin et al., 2020; Ricke et al., 2020). In general, the antimicrobial properties of organic acids likely arise from lower pH affecting enzymatic transport systems and energy generation (Dittoe et al., 2018; Khan et al., 2022). However, a number of organic acids were not identified in our screen, suggesting that some may be more inhibitory than others. Some organic acids even significantly improved desiccation survival, such as α-keto-glutaric acid and d-gluconic acid (Supplementary Table S1). The mechanisms of how specific organic acids affect survival during *Salmonella* surface colonization should be studied further.

We identified an inhibitory effect by deoxyribose that is commonly found as the sugar component in DNA, although the chemical was not expected to be detrimental to *Salmonella*. Similarly, guanine, one of the main nucleobases found in DNA and RNA, was also identified as an inhibitory biomolecule. Alloxan is both a pyrimidine derivative and a glucose analog, suggesting that it could be acting either through a nucleotide-based mechanism or by inhibiting glycolysis or another glucose-linked pathway (Lenzen, 2008). Dihydroxyacetone is a triose whose phosphorylated variant (DHAP) is a component of the glycolysis/gluconeogenesis pathway (Orozco et al., 2020). These compounds may be instituting a signaling response to extracellular carbohydrates and nucleotides to dysregulate surface survival, or the biomolecules may be interfering with appropriate carbohydrate and nucleotide metabolic pathways within the bacterium. Future understanding of how these inhibit *S.* Typhimurium surface colonization may uncover other metabolic and signaling inhibitors worth further study.

Of the biomolecules identified, salicylic acid possessed both low minimal (62.5 μM) and low complete inhibitory concentrations (250 μM). Moreover, salicylic acid was also shown to inhibit *Salmonella* colonization of an organic surface in the eggshell. The possible mechanism as to why salicylic acid is toxic to surface-colonizing *Salmonella* is unclear. In addition to the possibility of a pH-related inhibitory activity by this organic acid as described above, we had also previously identified that salicylic acid reduced *S.* Typhimurium cellulose production (Mills et al., 2015), suggesting that it may have a signaling effect within the bacterium. While we were unable to find a role for cellulose production in eggshell colonization, salicylic acid may be signaling to the bacterium in such a way that prevents it from properly adapting to survival on a surface. Salicylic acid also serves as a crucial hormone in plant innate immunity, specifically the systemic acquired resistance response where local defense activation enhances immune responses in the entire plant (Huang et al., 2020). Raw leafy greens are a common source of *Salmonella-*associated illness, and *Salmonella* has been demonstrated to persist in lettuce, including through internal colonization of plant tissue (Jechalke et al., 2019). Therefore, *Salmonella* likely encounters salicylic acid to some degree when colonizing plants, and it then may turn on appropriate virulence genes to combat the plant immune system. Feasibly, the addition of salicylic acid on surfaces may cause *Salmonella* to turn on virulence genes that hinder surface survival through the exertion of unneeded energy in a nutrient-scarce environment.

Choi et al. (2018) reported that exposure to salicylic acid decreases the expression of OmpF, which is a major general diffusion porin that allows hydrophilic molecules into *Salmonella*, as is in other Gram-negative bacilli species such as *E. coli* and *S. marcescens*. Since the diffusion of nutrients would be vital in a nutrient-scarce environment such as surface colonization, salicylic acid decreasing expression of a diffusion porin may be detrimental to survival. Salicylic acid is also a potent inducer of the AcrAB efflux pump system, which can confer antimicrobial resistance to cephalosporins and fluoroquinolones (Hartog et al., 2010; Choi et al., 2018). Conceivably, exposure to salicylic acid on surfaces could permit the inappropriate expression of the efflux pump AcrAB, which could inadvertently pump out beneficial molecules. Upon a surface—an already nutrient-scarce scenario—this could hinder nutrient retainment.

This Phenotype Microarray plate screen has proven to be a useful format for screening nutrients for their effect on bacterial survival. We hope to screen additional compounds in the future using further Phenotype Microarray nutrient plates containing phosphorus and sulfur sources, which would introduce more chemical diversity into the screen already conducted. Furthermore, identified inhibitors should be tested for their ability to inhibit the colonization of non-plastic inorganic and organic surfaces, the stability of the inhibitor upon the surface, and their utility within agricultural settings. While our hope in using these nutrient-type biomolecules was to limit the potential toxicity upon poultry and humans within these facilities, detailed analysis of the toxic effects will be necessary prior to implementation. Although we have shown an inhibitory effect from our identified biomolecules, it is quite possible that any single biomolecule may not kill sufficiently within an agricultural setting. Instead, these may better serve as combinations either between multiple biomolecules or as an addition to compounds already in use to increase efficacy and reduce the incidence of resistance to a single inhibitor. Testing into which combinations exhibit the greatest reduction in these areas will help uncover the ideal commercial use. Finally, understanding the mechanisms behind how each potential inhibitor prevents surface colonization would be beneficial in identifying other compounds missed in this screen that could inhibit *Salmonella*, identify potential biomolecule combinations based on targeting multiple pathways, and give insight into possible common pathways to exploit for the broad-spectrum activity of these inhibitors.

To identify possible new therapeutics in the face of growing desiccation and antibiotic resistance, we proposed the use of nutrient-type biomolecules as a possible mechanism to find naturally occurring compounds that could disrupt *Salmonella* survival. This study offers the groundwork for creating new solutions in the fight against bacterial contamination in agriculture, addressing the problem at the source to aid in the prevention of increasingly drug-resistant pathogens that cause foodborne illness.

## Data Availability

The original contributions presented in the study are included in the article/Supplementary Material; further inquiries can be directed to the corresponding author.
